# Prevalence of smear positive pulmonary tuberculosis among outpatients presenting with cough of any duration in Shashogo Woreda, Southern Ethiopia

**DOI:** 10.1186/s12889-015-1411-4

**Published:** 2015-02-10

**Authors:** Endale Eliso, Girmay Medhin, Mulugeta Belay

**Affiliations:** Shashogo Woreda Health Office, Hadiya Zone, Hosanna Ethiopia; Aklilu Lemma Institute of Pathobiology, Addis Ababa University, P.O. Box 1176, Addis Ababa, Ethiopia

**Keywords:** Prevalence, Smear positive pulmonary TB, Cough less than 2 weeks, Southern Ethiopia

## Abstract

**Background:**

Excluding patients with cough less than two weeks from screening for TB which is the current practice of TB control program in Ethiopia may result in delayed diagnosis thereby increasing transmission risk to others. The current study aimed to determine the prevalence of smear positive pulmonary tuberculosis among patients presenting with cough to four health centers in Shashogo woreda, Southern Ethiopia.

**Methods:**

A cross sectional study was conducted in four health centers in Shashogo Woreda, between November 2011 and March 2012. Four-hundred and sixty one patients aged five years and above attending the outpatient clinics and reporting cough of any duration were screened for pulmonary TB using smear microscopy. During data analysis, patients were classified by duration of cough with the cut-off of two weeks. Stata version 11 was used for data analysis.

**Results:**

A total of 299 patients with cough of two or more weeks and 162 patients with cough less than 2 weeks were recruited. The overall prevalence of smear positive pulmonary TB was 4.6% (95% CI: 2.6% to 7.7%). The prevalence of smear positive pulmonary TB among patients with cough lasting two or more weeks was significantly higher compared to those patients with cough lasting less than two weeks (6.0% versus 1.9%; p = 0.04).

**Conclusion:**

Although the prevalence of smear positive pulmonary TB among patients with cough less than 2 weeks was low, considering the contribution of delayed diagnosis for continued transmission of TB, screening patients with cough less than 2 weeks might be considered for TB control. A multi-site study with large sample size is needed to substantiate the current findings.

## Background

Tuberculosis (TB) is a major cause of morbidity and mortality worldwide. In 2012 alone, there were 8.6 million new cases and 1.3 million deaths globally [1]. Twenty years have passed since World Health Organization (WHO) declared TB as a global public health emergency. To this end, significant progress has been made towards achieving the 2015 global targets within the framework of the Millennium Development Goals [1]. However, WHO African and European Region are off-track in reducing mortality and prevalence [1]. While a high treatment success has been achieved using Directly Observed Treatment, Short Course (DOTS), low case detection rate remains an obstacle to the long term success of TB control programs in TB prevalent settings [2].

According to the WHO Global TB report, Ethiopia ranks seventh among the 22 TB high burden countries in the world and third in Africa behind Nigeria and South Africa [1]. TB is the leading cause of morbidity, the second cause of death and the third cause of hospital admissions in Ethiopia [3]. A recent population-based prevalence survey in Ethiopia indicated a lower (108/100,000) prevalence of smear positive pulmonary TB (PTB) than previously estimated [4] probably indicating a good program performance. However, a large proportion of TB cases was among the young population suggesting that TB is circulating in the community.

In order to curb the impacts of TB, the government has carried out various interventions including fully integrating TB diagnosis and treatment into the general health service system and decentralizing TB service delivery to the peripheral health units at Woreda level. The DOTS strategy has been incorporated in the TB prevention and control program since 1992 [3] and the geographic coverage is reaching nearly 100% [5] with treatment success rate of 84%. However, case detection rate of new smear positive cases is 28% which is far below the WHO target [6].

Previous studies [7,8] elsewhere have reported that a significant proportion of TB cases were diagnosed among patients presenting with cough less than 2 weeks. Currently, screening for TB in Ethiopia is limited to patients presenting with cough lasting at least 2 weeks. On the other hand, a long delay in the diagnosis of TB is a well documented problem in the management of TB [9-11]. Excluding patients with cough lasting less than 2 weeks might lead to delayed diagnosis and continued transmission. In this study, we estimated the prevalence of smear positive pulmonary TB among patients with cough of any duration in the outpatient clinics of four health centers in Shashogo woreda, Southern Ethiopia.

## Methods

### Setting and study participants

Administratively, Ethiopia is divided into 9 regional states and two City administrations. Each administrative Region is divided into several Zones and each Zone is divided into several Woredas (the third level administrative divisions). The health service delivery system also follows the political administrative structure. Each Woreda has health centers in such a way that 25,000 people are served in one health center.

Data collection for the current study was done between November 2011 and March 2012. A cross-sectional study design was used to recruit patients with cough of any duration at the outpatient clinics of four health centers in Shashogo Woreda, Hadiya Zone, Southern Nations, Nationalities, and Peoples' Region of Ethiopia. The administrative Capital of Hadiya Zone is Hosanna and there are five health centers in Shashogo Woreda. The four health centers included for patient recruitment are Bonosha, Jemaya, Hirko & Shemo. The fifth health center was excluded from the current study since it was not providing smear microscopy service during the study period.

Sample size required in each group of patients (i.e. patients with cough lasting less than 2 weeks and patients with cough lasting for at least two weeks) was estimated using a method appropraite to estimate a single population proportion [12]. We assumed a 14.6% prevalence of smear positive PTB among patients with cough of two weeks [13] and 7.1% prevalence (i.e. half the prevalence among those with cough lasting at least 2 weeks) of smear positive PTB among patients with cough less than two weeks, 4% margin of error in the estimates in each group and 95% confidence level. With these assumptions, the required sample sizes were 299 for those with cough lasting two or more weeks and 162 for those with cough lasting less than two weeks. Hence, a total of 461 patients presenting to the outpatient clinic of the target health centers with cough of any duration were included in this study.

### Data collection, smear microscopy and quality control

Before data collection, health workers who have been working at the outpatient departments of the four health centers were trained. The health workers registered patients aged five years and above with cough of any duration and requested patients to submit three sputum samples as per the national guidelines [3]. Data on patients’ marital status, occupation, education, duration of cough in days or weeks and sputum results were collected. One focal person from each health center out-patient case team was given responsibility of coordinating data collection. Since the standard procedure for diagnosis of pulmonary tuberculosis in Ethiopia is through passive case finding, where all patients with cough for two or more weeks are required to submit three sputum samples in the form of “spot-morning-spot” [3], the same procedure was used in this study. Training was given to all laboratory personnel from the four health centers to harmonize smear microscopy procedures. The quality check for the submitted samples was done according to the national guideline [14]. External quality assessment as well as on-site evaluation of methods and procedures was done at each peripheral health facility laboratory level. During the study period, Hossana sub-regional laboratory conducted quality assurance as part of its routine external quality assessment. All positive and some negative slides were re-checked blindly (blinded re-checking) for quality control and the results were satisfactory as confirmed by the sub-regional laboratory.

Besides, to evaluate the trends of smear positive pulmonary TB and to compare the result with the current prevalence, we extracted secondary data from laboratory registration books of each health center covering a period of six years.

### Data analysis

Data was computerized using EpiData version 3.1 and data analysis was performed using Stata version 11. The proportion of patients with smear positive PTB was calculated for the two study groups (i.e. according to their cough duration). A possible association between PTB and patients’ background characterstics as well as cough duration was investigated using Pearson’s chi-square. Statistically significant associations were reported whenever p-value was less than 5%.

### Ethical considerations

The study was approved by the Institutional Review Board of Aklillu Lemma Institute of Pathobiology, Addis Ababa University before commencing data collection. Written consent was signed by each participant (guardians for children) before enrolment into the study. All patients with smear positive PTB were referred to the TB clinic for treatment and smear negative patients were treated according to the National Guideline.

## Results

### Background characteristics of study participants and their association with smear results

During the study period, a total of 461 patients with cough were screened for PTB using AFB staining and smear microscopy. Socio-demographic characteristics of study participants and their association with smear result is summarized in Tables 1 and 2. The minimum and the maximum ages were 5 years and 85 years, respectively, and the mean age was 32.4 (SD = 14.8) years. Among the study participants, 41.0% were in the age range of 25–39 years, 9.3% were below the age of 14 years, 46.2% were females and 71.1% were married.

**Table 1:** **Associations of background characteristics with sputum smear results among OPD attendants with cough at Shashogo Woreda, Southern Ethiopia**

**Characteristics**	**Smear positive number**	**Smear negative number**	**p-value**
**Sex**			
Male	10	203	0.89
Female	11	237	
**Age (in years)**			
5-14	0	43	0.29
15-24	3	83	
25-39	12	177	
≥40	6	137	
**Marital status**			
Married	16	312	0.60
Single	5	128	
**Occupation**			
Farmers	10	163	0.62
House wives	6	145	
Others	5	132	
**Education**			
No formal education	12	210	0.70
Elementary	7	176	
Secondary & higher	2	54	
**Religion**			
Protestants	11	260	0.54
Islam & others	10	180	
**Ethnic group**			
Hadiya	16	351	0.12
Silite	3	69	
kembata	1	18	
Others	1	2	
**Cough duration**			
<2 weeks	3	159	0.04
≥2 weeks	18	281	
Location of HC*			
Urban	12	193	0.23
Rural	9	247	

*HC = Health center, OPD= outpatient department.

**Table 2:** **Associations of background characteristics with sputum smear results among patients with cough less than 2 weeks and those with cough of 2 weeks or more in Shashogo Woreda, Southern Ethiopia**

**Characteristics**	**Categories**	**Total examined number (%)**	**Cough < 2 weeksSmear positive, number (%)**	**Cough ≥ 2 weeksSmear positive, number (%)**	**p-value**
**Sex**	Male	213 (46.2)	1 (1.4)	9 (6.4)	
	Female	248 (53.8)	2 (2.2)	9 (5.7)	0.89
**Age (years)**	5-14	43 (9.3)	0 (0.0)	0 (0.0)	
	15-24	86 (18.6)	0 (0.0)	3 (5.7)	0.29
	25-39	189 (41.0)	3 (4.0)	9 (7.9)	
	≥40	143 (31.0)	0 (0.0)	6 (6.0)	
**Marital status**	Married	328 (71.0)	2 (1.8)	4 (4.8)	
	Single	133 (29.0)	1 (2.0)	14 (6.5)	0.60
**Occupation**	Farmer	173 (37.5)	2 (3.3)	8 (7.1)	
	House wive	151 (32.8)	1 (1.85)	5 (5.2)	0.62
	Students & others	137 (29.7)	0 (0.00)	5 (5.6)	
**Education**	None	222 (48.0)	1 (1.4)	11 (7.4)	
	Elementary	183 (40.0)	1 (1.6)	6 (5.0)	0.70
	Secondary and above	56 (12.0)	1 (4.0)	1 (3.2)	
**Religion**	Protestant	271 (58.8)	1 (1.0)	10 (5.8)	
	Islam and others	190 (41.2)	2 (3.1)	8 (6.3)	0.54
**Ethnic group**	Hadiya	367 (79.6)	2 (1.6)	14 (5.8)	
	Silite	72 (15.6)	0 (0.0)	3 (6.5)	0.58
	Others	22 (4.8)	1 (8.3)	1 (10)	
Number of symptoms*	1 – 4	171 (37.0)	2 (2.74)	0 (0.0)	
	>4	290 (63.0)	1 (1.12)	18 (8.96)	0.007

*cough, fever, poor appetite, feeling weak, chest pain, night sweats, weight loss, shortness of breathing.

Although the majority of the study participants were males, there was no significant association between gender and duration of cough (categorized as < 2 weeks or ≥ 2 weeks) (p = 0.58). A total of 21 patients were smear positive and the overall prevalence of smear positive PTB was 4.6%. Among patients who coughed for two weeks or more, 6.0% (95% CI: 3.6% to 9.3%) had smear positive PTB. Similarly, among those who had cough for less than two weeks, 1.9% (95% CI: 0.4% to 5.3%) had smear positive PTB. The prevalence of smear positive PTB among patients with cough lasting at least 2 weeks was significantly higher compared to those patients presented with cough less than 2 weeks (p = 0.04) (Table 1). All smear positive PTB patients among those with cough of 2 weeks or more had more than 4 symptoms whereas only 1 of the 3 smear positive PTB patients among those with cough less than 2 weeks had more than 4 symptoms and the difference was statistically significant (p = 0.007) (Table 2).

### Results from record review

**Figure 1:**
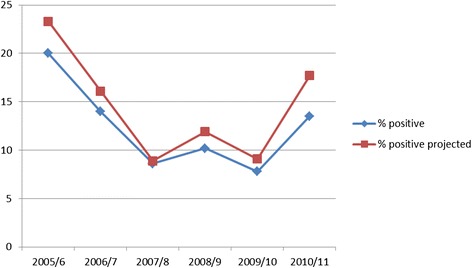
**Trends of smear positive PTB among PTB suspected attendants at the four selected health centers, Shashogo Woreda,Southern Ethiopia.** Blue color line with squares indicates percentage of smear positive PTB among all screened each year. Red color line with squares indicates projected percentage of smear positive PTB among all screened each year if those with cough less than 2 weeks were screened for PTB. X-axis represents time in year and y-axis represents percentages of smear positive PTB.

Medical records of 1273 patients which were registered in the TB laboratory registration books of the four study health facilities over 6 years (July 2005 to June 2011) were reviewed. The focus of the document review was on the smear positive PTB patients among those with smear examination results recorded in the health facilities. The result showed that among patients with cough with smear examination results over the 6 years period, 11.0% (141/1273) were smear positive and smear positivity was much higher in July 2005-June 2006 (20%) and lowest in the year July 2009-June 2010 (7.8%) (Figure 1).

## Discussion

In this study, the overall prevalence of smear positive PTB was 4.6%. The prevalence of PTB among those with cough lasting 2 weeks was 6.0% whereas the corresponding prevalence among those with cough less than 2 weeks was 1.9%. The result showed that prevalence of smear positive PTB among patients with cough ≥ 2 weeks was much lower than the prevalence reported in different parts of Ethiopia [10,13,15-17] and elsewhere [7]. However, the prevalence in this study was comparable to the prevalence reported in another study in South-West Ethiopia [18]. The lower prevalence in this study compared with the prevalence in other studies may partly be explained by the fact that all facilities included in this study were primary health care facilities where most of the patients with cough were detected early which might have increased the denominator population. Supporting this view, a study from Tanzania showed a significant difference in smear positivity among attendants with cough lasting 2 weeks or more between primary (7.7%) and secondary health care facilities (14.5%) [7]. This suggests that a higher number of suspects are examined with a lower smear positive rate at primary health care facilities as previously observed [18]. Another possible explanation is that existing TB programme has been able to effectively detect and treat most of the infectious TB cases and that the incidence of the disease is on the decline in the setting as previously reported [19,20]. This is also supported by the trend of smear positive pulmonary TB over 6 years in the current study.

We found a significant difference in the prevalence of smear positive PTB among patients who coughed for less than two weeks and those who coughed for two weeks or more. This is in agreement with a previous study in Peru in which smear positivity was significantly lower among those with short duration of cough compared to those with long duration of cough [21]. However, our finding contradicts the results of another study in Tanzania which reported comparable prevalence of smear positive PTB among those with short duration of cough and those with long duration of cough [7]. Unlike our study, the study in Tanzania included secondary health care facilities (hospitals) from which a comparable prevalence of PTB in those with short and long duration of cough was reported; however, for low level health facilities, similar to our finding, the study from Tanzania reported a lower prevalence of PTB in those with short duration of cough compared to those with long duration of cough [7].

The prevalence of smear positive PTB among those who coughed less than 2 weeks is significantly lower compared to those with cough lasting 2 weeks or more. Besides, the absolute number of smear positive PTB patients among those with cough less than 2 weeks was small partly because of the small sample size. Even then, 2 cases could be missed among 100 patients with cough less than 2 weeks. However, our results need to be substantiated with an independent study with a large sample size. Delayed diagnosis is an important problem in the management and control of TB [9] and smear positive PTB patients with cough less than 2 weeks could be reluctant to report again if they are returned with reassurance or antibiotics. This may contribute to delayed diagnosis and continued transmission which obviously would affect TB control efforts. Weighing the economic cost of screening all patients with cough against the impact of continued transmission on TB control as a result of delayed diagnosis is important. Alternatively, developing clinical algorithm to screen out those with other respiratory illnesses could be considered as suggested previously [8].

Unlike previous studies, this study looked into the prevalence of smear positive PTB among those with cough less than 2 weeks to investigate if we are missing smear positive PTB patients because of the 2 weeks cut-off for PTB screening. However, it did not try to look for diagnostic algorithm that would identify TB patients among those patients with cough less than two weeks. Moreover, the sample size is small considering the low prevalence of smear positive PTB. Finally, the work load and feasibility of screening all patients with cough was not evaluated.

## Conclusions

Prevalence of smear positive PTB among patients who coughed for less than two weeks was significantly lower compared to the prevalence among those who coughed for at least two weeks. However, considering the fact that diagnostic delay is a serious problem, the proportion of pulmonary TB among patients with cough less than 2 weeks cannot be ignored. A multi-site study with large sample size aiming at investigating the prevalence of TB among patients with cough less than 2 weeks is needed.

## References

[1] WHO. Global tuberculosis report 2013. Geneva; 2013. http://apps.who.int/iris/bitstream/10665/91355/1/9789241564656_eng.pdf. Accessed on 12 August 2014.

[2] Xianyi C, Fengzeng Z, Hongjin D, Liya W, Lixia W, Xin D (2002). The DOTS strategy in China: results and lessons after 10 years. Bull World Health Organ.

[3] MOH. Tuberculosis, leprosy and TB/HIV prevention and control programme. MANUAL. 4^th^ Edition. Addis Ababa; 2008. http://www.who.int/hiv/pub/guidelines/ethiopia_tb.pdf. Accessed on 12 August 2014.

[4] Kebede A, Alebachew Z, Tsegaye F, Lemma E, Onozaki I, Sismanidis C (2014). The first population-based national tuberculosis prevalence survey in Ethiopia, 2010–2011. Int J Tuberc Lung Dis.

[5] WHO. Global Tuberculosis Control | WHO REPORT 2008: Ethiopia, country profile. Geneva; 2008. http://www.stoptb.org/assets/documents/countries/acsm/Ethiopia.pdf. Accessed 20 January 2015.

[6] WHO. Global tuberculosis control report 2009. Geneva; 2009. http://whqlibdoc.who.int/publications/2009/9789241563802_eng_doc.pdf. Accessed on 12 August 2014.

[7] Ngadaya ES, Mfinanga GS, Wandwalo ER, Morkve O (2009). Detection of pulmonary tuberculosis among patients with cough attending outpatient departments in Dar Es Salaam, Tanzania: does duration of cough matter?. BMC Health Serv Res..

[8] Banda HT, Harries AD, Welby S, Boeree MJ, Wirima JJ, Subramanyam VR (1998). Prevalence of tuberculosis in TB suspects with short duration of cough. Trans R Soc Trop Med Hyg.

[9] Belay M, Bjune G, Ameni G, Abebe F (2012). Diagnostic and treatment delay among tuberculosis patients in Afar Region, Ethiopia: a cross-sectional study. BMC Public Health.

[10] Demissie M, Lindtjorn B, Berhane Y (2002). Patient and health service delay in the diagnosis of pulmonary tuberculosis in Ethiopia. BMC Public Health.

[11] Yimer S, Bjune G, Alene G (2005). Diagnostic and treatment delay among pulmonary tuberculosis patients in Ethiopia: a cross sectional study. BMC Infect Dis.

[12] Lwanga SK, Lemeshow S (1991). Sample size determination in health studies.

[13] Yohanes A, Abera S, Ali S (2012). Smear positive pulmonary tuberculosis among suspected patients attending Metehara sugar factory hospital, Eastern Ethiopia. Afr J Health Sci.

[14] WHO. Laboratory services in tuberculosis control: microscopy. Part II. Geneva; 1998. (http://whqlibdoc.who.int/hq/1998/WHO_TB_98.258_%28part2%29.pdf).

[15] Deribew A, Negussu N, Melaku Z, Deribe K (2011). Investigation outcomes of tuberculosis suspects in the health centers of Addis Ababa. Ethiopia PloS one.

[16] Gebre D, Mimano LN (2010). Prevalence of smear positive pulmonary tuberculosis among patients attending Seka Health Center, Jimma, Oromia Region, Ethiopia. East Afr J Public Health.

[17] Bruchfeld J, Aderaye G, Palme IB, Bjorvatn B, Britton S, Feleke Y (2002). Evaluation of outpatients with suspected pulmonary tuberculosis in a high HIV prevalence setting in Ethiopia: clinical, diagnostic and epidemiological characteristics. Scand J Infect Dis.

[18] Ali H, Zeynudin A, Mekonnen A, Abera S, Ali S (2012). Smear positive pulmonary tuberculosis (PTB) prevalence amongst patients at agaro teaching health center, South West Ethiopia. Ethiop J Health Sci.

[19] Shargie EB, Lindtjorn B (2005). DOTS improves treatment outcomes and service coverage for tuberculosis in South Ethiopia: a retrospective trend analysis. BMC Public Health.

[20] Shargie EB, Yassin MA, Lindtjorn B (2006). Prevalence of smear-positive pulmonary tuberculosis in a rural district of Ethiopia. Int J Tuberc Lung Dis.

[21] Otero L, Ugaz R, Dieltiens G, Gonzalez E, Verdonck K, Seas C (2010). Duration of cough, TB suspects’ characteristics and service factors determine the yield of smear microscopy. Trop Med Int Health.

